# Identification of aquifer pollution’s point sources with the reciprocity principle

**DOI:** 10.1038/s41598-022-13795-w

**Published:** 2022-06-15

**Authors:** Rachida Bouhlila, Nejla T. Hariga

**Affiliations:** 1 ENIT, Université de Tunis El Manar, Tunis, Tunisia; 2 INAT, Université de Carthage, Tunis, Tunisia

**Keywords:** Environmental sciences, Hydrology, Engineering, Mathematics and computing

## Abstract

The principle of reciprocity, called Maxwell–Betti theorem, initially used in mechanics in an elastic structure, establishes a relation of equality between two distinct strains under different loads. In this paper, we extend and apply this principle to flow and solute transport equations in porous media, in order to perform the pollution sources identification in aquifers. We developed general 2D expressions of the reciprocity principle for transient transport problems. This model leads to a linear equations set, with point sources coordinates, concentrations and associated water fluxes as unknowns The proposed model is then applied to the Rocky Mountain Arsenal aquifer (Konikow in Modeling Chloride Movement in the Alluvial Aquifer at the Rocky Mountain Arsenal, Colorado. Technical Report Water-Supply Paper 2044, USGS, 1979), where polluted water is injected into a well in the domain. The used inverse technique successfully recovered the position and the pollutant concentration in addition to the associated water flux. In addition, we developed and implemented the inverse method for different knowledge levels of the degrees of the aquifer contamination, i.e. more or less data available in the field. Multiple pollution point sources and noisy data situations are also developed and tested with high efficiency. The proposed method would be easy and useful to be implemented in the modeling software now widely used by researchers and groundwater managers. It can thus be applied in real case studies, to help authorities and regulators to efficiently identify the polluters and the contamination process, i.e. its location, onset, duration and the associated mass and water fluxes.

## Introduction

Nowadays, the industrial development and the intensification of the agricultural activities, introduce continuously in the environment new molecules, more or less carefully produced and used. This requires the utmost vigilance on behalf of the sanitary and water management authorities^1–3^. Indeed, a major part of the world population relies, to some extent, on groundwater for drinking, and for crops and food production. The protection of these resources should thus be of a great concern.

For many years, it was believed that the layers of soil and sediment above an aquifer act as a natural filter that retains pollutants and thus protects the groundwater. However, it has been widely recognized that the capacity of these soil layers to retain pollutants can be exceeded very quickly^4^. In addition, since an aquifer is polluted, it may become unusable for decades. The remediation of contaminated groundwater is inherently complex and expensive and can require long periods of time and sometimes centuries^5^.

Actually, decontamination of polluted groundwater is a huge challenge in Hydrogeology, in relation with the environmental and health requirements. A main challenge in any rehabilitation action is the evaluation of the degree of contamination. This includes the identification of unknown sources of contamination and the corresponding water and solute fluxes that led to the present state of pollution.

This process is important for both understanding the implementation of adequate remedial measures and for the identification of causes and responsibilities. In fact, the identification of the location and the level of pollution sources is crucial for the application of the polluter-pay principle adopted by the United Nation Conference of Rio de Janeiro Environment and Development declaration in its article 16^6^. In addition, the contaminant source identification could also be a means of dissuading potential infringements of laws for pollutant discharge and waste repository managements.

In this context, many works have considered this inverse problem in hydrogeology. Most of the existing studies concerns the recovering of the points-sources locations and/or the contaminant release histories^7,8^ and/or the number of these points-sources^9^.

Atmadja and Bagtzoglou^10^ presented a review on mathematical methods that have been developed for the study of identifying sources of contamination. Authors classified these methods as deterministic or stochastic and associated to an optimization model^8,11–17^. Other heuristic approaches based on genetic algorithm are proposed in Refs.^9,11^.

In this paper, we introduce a new method based on the reciprocity principle, that allows the simultaneous identification of pollution point-sources locations and their pollutants concentrations, from the concentration’s measurements in the aquifer domain. This principle, also known as the Maxwell–Betti theorem^18^, was first introduced in mechanics for linear problems^19^. It stipulates that for a linear elastic structure subject to two forces F and G, the work resulting from the application of the force F on the displacement field, yielded by the force G, is equal to the work resulting from the application of the force G on the displacement field yielded by the force F.

From a phenomenological perspective, this principle establishes strong relationships between different sets of forces and the consequent displacements applied to a given structure.

In mechanics, the reciprocity principle is usually applied to obtain displacements due to complex forces by using proxy problems with simpler forces that are more easily solved. Within the framework of groundwater flow scenario, reciprocity between two interference pumping tests was analyzed by Bruggeman^20^ for Darcian flows in an unbounded, heterogeneous porous medium. Hariga et al.^21–24^ have also applied the reciprocity principle in groundwater flows by using sources and boundary conditions as forcing terms and the resulting head field as a consequence.

In this research, the reciprocity principle is applied to the transport equation to recover the features of the pollutant point sources in aquifers. We show that this method allows to evaluate the position, solutes concentration and the associated injected water flux, for point solutes sources, from the knowledge of the cumulative mass flux through the boundary at any time in the considered interval and the concentrations on all the domain at the given time. The proposed method can then be considered as a “direct” one according to the Neuman classification^25^.

Obviously, in the real-world scenarios of contamination problems, since the concentrations are measured in a finite number of points, these should first be interpolated throughout the considered domain.

We show that the accuracy of the present identification method, depends on the number of available data. However, reasonable results are also obtained with few data which is a valuable insight to guide managers in the process of pollution sources identification.

The remainder of this paper is structured as follows. After a general review of the reciprocity principle (“General formulation and interpretation of the reciprocity principle” section), we derive it for the advection–diffusion equation with a constant injection during a given time (“The transport and flow equations in porous media” section). We then illustrate it in point-source pollution identification (“Illustration of the method for pollution sources identification” section) for four scenarii: with complete data, with few observations, with noisy data and with multiply point sources.

## The reciprocity principle applied to the advection–diffusion equation

### General formulation and interpretation of the reciprocity principle

Built on the Maxwell–Betti principle^18^, for the sake of simplicity we express the reciprocity principle in a general mathematical framework for linear elliptic problems. Let ${\mathcal {V}}$ be a Hilbert space associated to a domain $\varOmega$, ${\mathfrak {a}}$ a bilinear form on ${\mathcal {V}}$, assumed to be symmetric, continuous and coercive and ${\mathfrak {l}}_i$ a linear form defined on ${\mathcal {V}}$ for $i = 1,2$ assumed to be continuous. Then we define the following variational problem:

1 $$\begin{aligned} \begin{array}{cc} Find~u_i~on~{\mathcal {V}}~such~that&{\mathfrak {a}}(u_i,\varphi )~=~{\mathfrak {l}}_i(\varphi )~ for~all~\varphi ~\in ~{\mathcal {V}}. \end{array} \end{aligned}$$

With $(u_i,\varphi )$ successively equal to $(u_1,u_2)$ and $(u_2,u_1)$ and using the symmetry of operator ${\mathfrak {a}}$, the reciprocity principle can be expressed by the identity:

2 $$\begin{aligned} {\mathfrak {l}}_1(u_2)~=~{\mathfrak {l}}_2(u_1). \end{aligned}$$

The reciprocity principle is fundamentally derived from the symmetry and the bi-linearity of the form ${\mathfrak {a}}$. From a physical perspective, it relates the responses to different external and internal forcing terms (source/sink terms, boundary conditions) of a given phenomenon on a fixed structure represented by form ${\mathfrak {a}}$. Finally, the reciprocity principle is similar to Green’s second identity giving way to the boundary element methods.

The crucial point of the method is the relevant choice of the test functions. The test functions should ideally be closely related to the initial problem but should also lead to much simpler and, if possible, analytical solutions.

### The transport and flow equations in porous media

The advection–diffusion equation governing solute transport in a domain over a time interval [t0 , tf] is:

3 $$\begin{aligned} \left\{ \begin{array}{llllll} \omega \frac{\partial C}{\partial t} - div(\overline{{\overline{D}}}\text{ grad } C -VC) &{}=Q &{} \text{ in }&{} \varOmega ~~ \times [t_0~,~t_f],&{} &{} \\ (\overline{{\overline{D}}}\text{ grad } C -VC).n &{} = \varPhi _N &{}\text{ on }&{} \varGamma _N \times [t_0~,~t_f],&{} &{} \\ C &{}= C_D &{} \text{ on }&{} \varGamma _D \times [t_0~,~t_f],&{} &{} \\ C(x,y,t_0)&{}=C_0&{} \text{ in }&{} \varOmega ,&{} &{} \end{array} \right. \end{aligned}$$

with *C*(*x*, *y*, *t*), the solute concentration $[ML^{-3}]$; $\omega$ the aquifer porosity; $\overline{{\overline{D}}}$ the hydrodynamic diffusion-dispersion tensor $[L^2T^{-1}]$; V the Darcy velocity $[LT^{-1}]$; $C_D$ the prescribed concentration at the Dirichlet boundary $\varGamma _D$; $\varPhi _N$ the prescribed flux at the remaining Neuman boundary $\varGamma _N$; $C_0$ the initial concentration distribution and *Q* is the source term.

In this work, we consider the case of point-sources pollutant injected during a finite time, so that *Q* is expressed by the following equation:

4 $$\begin{aligned} Q=\sum _{i=1}^{i=N_p}Q_{si}C_{si}\delta (x-S_i)\varPi \bigg (\frac{t_i-\frac{l_i}{2}-t}{l_i}\bigg ), \end{aligned}$$

where $N_p$ is the number of pollutant point-sources; $Q_{si}$ the fluid volume flux rate at the $i\mathrm{{th}}$ point-source $[T^{-1}]$; $C_{si}$ the pollutant concentration of the injected water at the $i\mathrm{{th}}$ point-source $[ML^{-3}]$ which starts at instant $t_i$ and stops at $l_i+t_i$; so $l_i$ represents the time during which the pollutant is injected in the aquifer. $\delta (x-S_i)$ is the Dirac function which is non zero only in the point-source located at $S_i=(x_i,y_i)$ and $\varPi$ is a rectangular function defined as:

5 $$\begin{aligned} \varPi (t)=H(t+1)-H(t-1), \end{aligned}$$

with *H* the Heaviside step function.

The transport problem (3) is coupled with the groundwater flow model via the Darcy velocity:

6 $$\begin{aligned} V=-Tgrad(h). \end{aligned}$$

Equation (6) is an expression of Darcy’s law, integrated over the thickness of the aquifer to lead to the classical horizontal 2D representation of aquifers^26^. The term *T* represents the transmissivity and is equivalent to the integral of the hydraulic conductivity over the thickness of the aquifer under the Dupuit assumption.

In a stationary 2D case, the hydraulic head *h* is solution of the following problem:

7 $$\begin{aligned} \left\{ \begin{array}{llll} -div(T(x,y)grad(h)) &{}=\sum _{j=1}^{j=N_f}Q_{si}\delta (x-S_j) &{} \text{ in }&{} \varOmega , \\ Tgrad(h).n &{}= Q_N &{}\text{ on }&{} \varGamma '_N,\\ h &{}=h _D&{} \text{ on }&{} \varGamma '_D, \end{array} \right. \end{aligned}$$

with $N_f$ the number of flow point-source $(N_f>N_p)$; *T*(*x*, *y*) the transmissivity field, $Q_{sj}$ the fluid volume flux rate at the $j^{th}$ point-source $[T^{-1}]$, $h_D$ the prescribed heads at the flow Dirichlet boundary $\varGamma '_D$, $Q_N$ the prescribed flux at the remaining flow Neuman boundary $\varGamma '_N$ .

Note that systems (3) and (7) are coupled via the Darcy velocity (6) and that the pollutant point-sources form a sub-set of the pumping point-sources.

Note that the assumption of a steady state for a certain period of time is widely adopted in hydrogeology and hydrogeological modeling. It concerns the period during which we can consider that the aquifer is in a little disturbed regime, with inflows and outflows that balance each other. This often corresponds to the aquifer before its exploitation or with a still low exploitation level^26^. When building a hydrogeological model of a given aquifer, we search in the available database, the moment that corresponds to a significant increase or decrease in the exploitation and/or piezometer, apart from seasonal variations if any. This moment is then considered as the beginning of the transient regime. The inertia of the hydrogeological systems being such that this moment is expressed in year and the various fluxes entering/leaving the system are considered constant for the period of time from the infinite to this date. The transient regime then begins and the various fluxes, and more rarely the boundary conditions, are calculated for each period of time chosen according to the reactivity/inertia of the aquifer: often monthly or seasonal or even annual.

Let us recall that the considered *i*nverse problem’s unknowns are: $C_{si}$ the concentration of the pollutant released during the time interval $[t_i, t_i+l_i]$ and the corresponding point source’s position $S_i=(x_i,y_i)$. In the other hand we have hydraulic head’s measurements and pollution concentration’s measurements in some points of the domain $\varOmega$.

We hereafter establish the reciprocity expression for the transient transport equation in a generic 2D domain.

### Reciprocity principle with the advection–diffusion equation

The reciprocity principle can be applied to the advection–diffusion equation using test functions $\varphi$ that verify:

8 $$\begin{aligned} div(\overline{{\overline{D}}}grad(\phi )=0~~~~~~~~~~~~~~\text{ in }~~\varOmega . \end{aligned}$$

Multiplying the first equations of systems (3) by $\phi$ and integrating it over $\varOmega$ then applying two times Green’s first identity, lead to the following equations:

$$\begin{aligned} \begin{array}{l} \int _\varOmega \omega \frac{\partial C}{\partial t}\phi - \int _\varOmega div(\overline{{\overline{D}}}\text{ grad } C -VC)\phi ~~~\\ \quad =\sum _{i=1}^{i=N_p}\langle Q_{si}C_{si}\delta (x-S_i)\varPi \bigg (\frac{t_i-\frac{l_i}{2}-t}{l_i}\bigg ),\phi \rangle \\ \\ \frac{\partial }{\partial t}\int _\varOmega \omega C\phi + \int _{\varOmega } \overline{{\overline{D}}}\text{ grad } C\text{ grad } \phi -\int _{\partial \varOmega }\overline{{\overline{D}}}\text{ grad }C.n \phi -\int _\varOmega VC\text{ grad }\phi \\ \qquad + \int _{\partial \varOmega } CV.n\phi ~~=~~~\sum _{i=1}^{i=N_p}Q_{si}C_{si}\phi (S_i)\varPi \bigg (\frac{t_i-\frac{l_i}{2}-t}{l_i}\bigg ) \\ \\ \frac{\partial }{\partial t}\int _\varOmega \omega C\phi -\int _{\partial \varOmega }(\overline{{\overline{D}}}\text{ grad }C - CV).n \phi + \int _{\partial \varOmega } \overline{{\overline{D}}}CV\text{ grad } \phi .n~\\ \qquad -\int _\varOmega VC\text{ grad }\phi = ~~\sum _{i=1}^{i=N_p}Q_{si}C_{si}\phi (S_i)\varPi \bigg (\frac{t_i-\frac{l_i}{2}-t}{l_i}\bigg ).\\ \end{array} \end{aligned}$$

Then, using the fact that the test function verify equation (8), leads to:

9 $$\begin{aligned} \begin{array}{lll} \frac{\partial }{\partial t}\int _\varOmega \omega C\phi -\int _{\partial \varOmega }(\overline{{\overline{D}}}\text{ grad }C - CV).n \phi + \int _{\partial \varOmega } \overline{{\overline{D}}}C\text{ grad } \phi .n \\ ~~~~-\int _\varOmega VC\text{ grad }\phi = \sum _{i=1}^{i=N_p}Q_{si}C_{si}\phi (S_i)\varPi \bigg (\frac{t_i-\frac{l_i}{2}-t}{l_i}\bigg )&{}&{} \end{array}. \end{aligned}$$

Integrating Eq. (9) over the time range $[t_0 , t_f]$ leads to the expression of the reciprocity principle for the advection–diffusion equation :

10 $$\begin{aligned} \begin{array}{l} \int _\varOmega \omega (C_f-C_0)\phi -\int _{t_0}^{t_f}\int _\varOmega VC\text{ grad }\phi -\int _{t_0}^{t_f}\int _{\partial \varOmega }(\overline{{\overline{D}}}\text{ grad }C - CV).n \phi \\ ~+\int _{t_0}^{t_f}\int _{\partial \varOmega } \overline{{\overline{D}}}C\text{ grad } \phi .n = ~\sum _{i=1}^{i=N_p}Q_{si}C_{si}\phi (S_i)\int _{t_i}^{t_i+l_i}\varPi \bigg (\frac{t_i-\frac{l_i}{2}-t}{l_i}\bigg )d\tau \end{array}, \end{aligned}$$

where $$C_f=C(x,y,t_f)$$.

Note that for a given test function $\varphi$, the left hand side of Eq. (10) is known. Then we can determine the point-source concentrations ($C_{si}$) and locations ($x_i,y_i$) by solving a linear system constitutes with Eq. (10) with different functions $\varphi$, as we will show it in the next example.

So in conclusion, the reciprocity principle relates the concentration and flux values at the boundary, the concentration values in the domain at any time with the pollution point-sources parameters.

However, since the wells water flow are also the pollutant point-source injection and as the flow equations are steady ones, it will be easier to apply the reciprocity principle to the system (7) as a first step of the identification procedure to recover the wells position (see^21–23^). Then the Eq. (10) is exploited to recover the pollutant concentration. This identification process will be illustrated in the following examples.

## Illustration of the method for pollution sources identification

First, we check the methodology with a single point-source pollution in the case of a hydrogeologic configuration inspired from the work by Konikow^27^ who predicted long term pollutants dispersion in groundwater flow due to a leaky chemical pond under the Rocky Mountain arsenal in Colorado, US. The model setup is directly inspired from a test case in SUTRA code developed by Voss^28,29^.

In 1984 Voss considers the Rocky Mountain Arsenal to demonstrate some of the capabilities of SUTRA software modelling^28^. This example serves him to demonstrate the applicability of SUTRA to an areal constant density solute transport problem.

Here, in our study, we think that this case is perfectly suited to our objectives insofar as it includes production and injection wells, all the characteristics of which must be found by the inverse method developed in this work.

**Figure 1:**
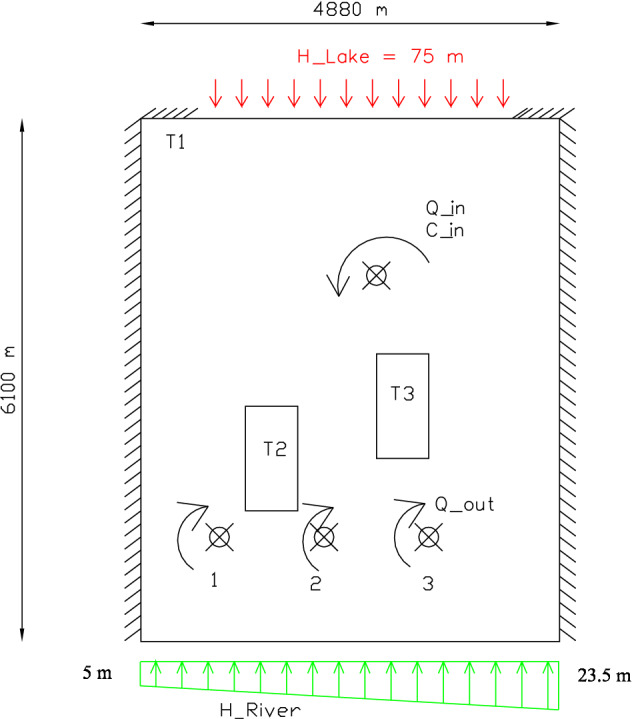
Rocky Mountain aquifer proprieties (where T1, T2 and T3 are the respective domains’ transmissivity).

We considered the same types of boundary conditions as the original problem and the geometric and hydrodynamic data used are those used in the works of Hariga et al.^21,23^. The results of the inverse calculation are therefore compared to those of the direct calculation for the chosen dataset. The porous media is supposed with isotropic heterogeneous properties. The geometry, with boundary conditions data, is sketched on Fig. 1. The domain is a 6100 m per 4880 m heterogeneous rectangle with a porosity $\omega = 0.2$, a transmissivity $T = 2.5 \times 10^{-4 }\,{\text {m}}^2/{\text {s}}$ and two less permeable zones of $T= 2.5 \times 10^{-8}\, {\text {m}}^2/{\text {s}}$. The aquifer is bounded upstream by a lake at the north with a constant head $h_n=75\, {\text {m}}$, a river downstream at the south with linear head, varying from 5 to 23.5 m and two impervious lateral borders. The aquifer is exploited by three (1, 2 and 3) pumping wells with a volumetric fluxes $Q_{out}^{1,3}=- \,0.008 \times 10^{-2}\, {\text {m}}^3/{\text {s}}$ and $Q_{out}^2=-\,0.016 \times 10^{-2} \, {\text {m}}^3/{\text {s}}$. The longitudinal dispersivity is $\alpha _L=30\, \text {m}$ and the transversal dispersivity is $\alpha _ T=3m$ in the entire domain.

**Figure 2:**
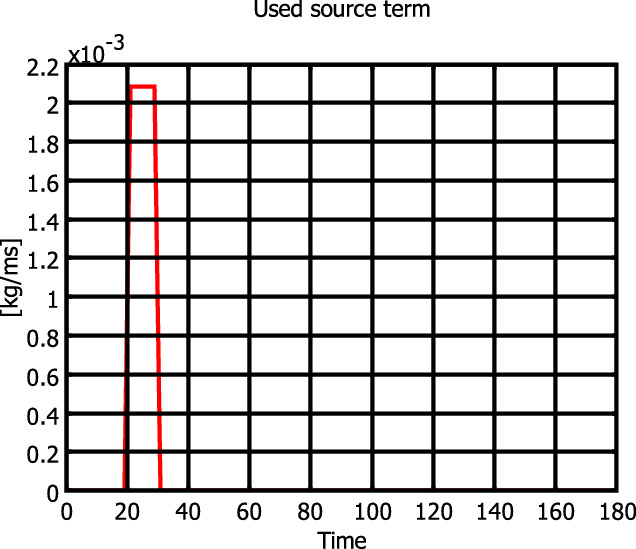
The used source term in Eq. (4).

Solute transport boundary conditions are zero prescribed concentration on upstream of the lake border and zero convective-dispersive flux on the lateral impervious border. A constant concentration ($C_s=1\, {\text {kg/m}}^3$) is assigned to the injected water at the contaminated pond, situated at (2745 m, 4270 m), with a water flux of $Q_{in}^4=0.002\, {\text {m}}^3/{\text {s}}$. We suppose that pollution injection starts at $t_1$ and continues for $l_1$ hours as shown on Fig. 2. The initial concentration is null over the entire domain.

The coupled flow and transport equations are solved using the FEM software Comsol Multiphysics^30^. The domain is meshed into 652 linear triangular elements and 364 nodes. Integrals are numerically evaluated using the Trapeze numerical integration.

The objective is to find the injected concentration of pollution $C_s$ and the position ($x_s,y_s$) of the point source,i.e. the pond, from the piezometric heads and the concentrations in the domain and the concentration flux over the boundaries at different times. As the problem has three unknowns, the reciprocity method requires only three virtual fields $\varphi _1, \varphi _2 \,and\, \varphi _3$. We choose three simple polynomial functions:

$$\phi _1=1, \phi _2 = Real(x+iy) = x\, and \, \phi _3= Im (x+iy) = y.$$

Their derivatives in the direction of the normal to the boundary of the domain are given by:

$$\frac{\partial \phi _1}{\partial n} = 0, \frac{\partial \phi _2}{\partial n} = n_x\, and \, \frac{\partial \phi _3}{\partial n}= n_y.$$

where $n = (n_x,n_y)$ the outward normal to the boundary.

Applied to these test functions, Eq. (10) and using the fact that:

$$\int _{t_1}^{t_1+l_1}\varPi \bigg (\frac{t_i-\frac{l_i}{2}-t}{l_i}\bigg )d\tau = l_1$$

leads to the following equations:

11 $$\begin{aligned}{}&\begin{array}{l} \int _\varOmega \omega C_f -\int _{t_0}^{t_f}\int _{\partial \varOmega }(\overline{{\overline{D}}}\text{ grad }C - CV).n ~~~~ =~~ 2l_{1}Q_{in}^4C_{s} \end{array} \end{aligned}$$

12 $$\begin{aligned}{}&\begin{array}{l} \int _\varOmega \omega C_f.x -\int _{t_0}^{t_f}\int _\varOmega VC.\text{ grad }\phi -\int _{t_0}^{t_f}\int _{\partial \varOmega }(\overline{{\overline{D}}}\text{ grad }C - CV).n.x \\ \quad + \int _{t_0}^{t_f}\int _{\partial \varOmega } \overline{{\overline{D}}}C.n_x ~~=~~l_{1}Q_{in}^4.x_pC_{s} \end{array} \end{aligned}$$

13 $$\begin{aligned}{}&\begin{array}{l} \int _\varOmega \omega C_f.y -\int _{t_0}^{t_f}\int _\varOmega VC.\text{ grad }\phi -\int _{t_0}^{t_f}\int _{\partial \varOmega }(\overline{{\overline{D}}}\text{ grad }C - CV).n.y +\\ \quad +~~\int _{t_0}^{t_f}\int _{\partial \varOmega } \overline{{\overline{D}}}C.n_y ~~=~~l_{1}Q_{in}^4.y_pC_{s} \end{array}. \end{aligned}$$

From Eq. (11), we note that we directly obtain the injected concentration $C_s$ from the knowledge of the cumulative mass flux through the boundary at any time in the interval [$t_0 , t_f$] and the concentration on all the domain $\varOmega$ at the final time $t_f$. Then we replace this value in Eq. (12) to obtain $x_p$ and in Eq. (13) in order to obtain $y_p$.

In Eqs. (12) and (13), we need to have the concentrations field at each time to identify the point-source position. However, as in this example, the pollution point-source is also a source point for the flow equation, we can apply the reciprocity principle to the *stationnary* problem (7) as done by Hariga et al in^21–23^ to identify the point-source position. It’s more easy and it consists in multiplying the first equation of system (7) by simple test functions ($\phi _1=x$ and $\phi _2=y$ ) and then using the Green formula twice times. So that at the end, we find the following expression for $x_p$ and $y_p$:

14 $$\begin{aligned} \begin{array}{lll} x_p &{}=&{} \frac{1}{Q_{in}^4}~\int _{\partial \varOmega } T(h.n_x -x.grad(h).n)\\ y_p &{}=&{} \frac{1}{Q_{in}^4}~\int _{\partial \varOmega } T(h.n_y -y.grad(h).n)\\ \end{array}. \end{aligned}$$

For the numerical study we define the relative errors as:

15 $$\begin{aligned} \epsilon _S = \frac{\Vert S_{exact}-S_{compute}\Vert _{L^2}}{\Vert S_{exact}\Vert _{L^2}}, \end{aligned}$$

for position’s identification, where *S* the vector position and $\Vert ~\Vert _{L^2}$ the euclidean norm.

And:

16 $$\begin{aligned} \epsilon _f= \frac{\Vert f_{exact}-f_{compute}\Vert _{L^1}}{\Vert f_{exact}\Vert _{L^1}}, \end{aligned}$$

for fluxes’ identification, where *f* is the injected pollution’s concentration and $\Vert ~\Vert _{L^1}$ the absolute value.

### Identification with complete data over the domain

For the injected concentration recovering we use Eq. (11), whereas for the point-source position identification we compare the two methodologies: the transport one by the use of Eqs. (12) and (13) and the stationary flow one by applying Eq. (14). We consider $t_1=25$ days and $l_1=5$ days.

**Figure 3:**
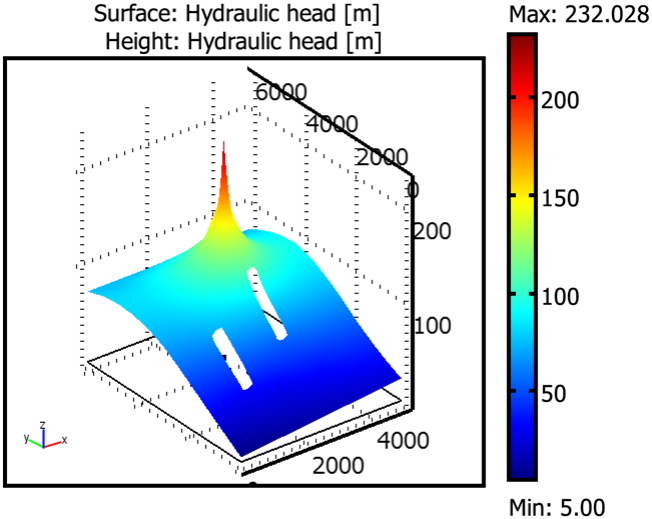
Rocky Mountain aquifer hydraulic head distribution.

**Figure 4:**
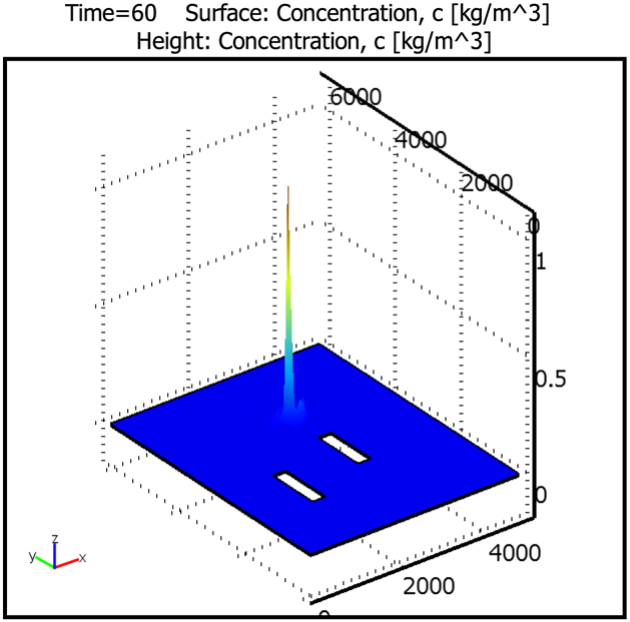
Rocky Mountain pollute concentration distribution.

**Figure 5:**
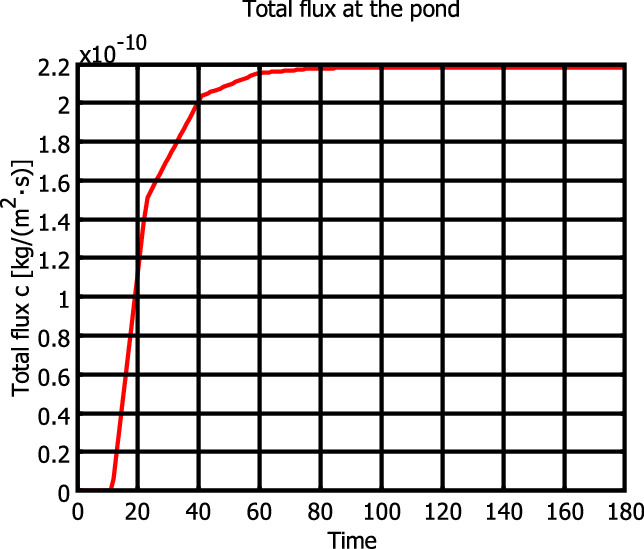
Total flux’s pollute distribution at the pond.

Figures 3, 4 and 5 show, respectively, the hydraulic head, the concentration distribution and the total flux at the pond over the domain.

**Figure 6:**
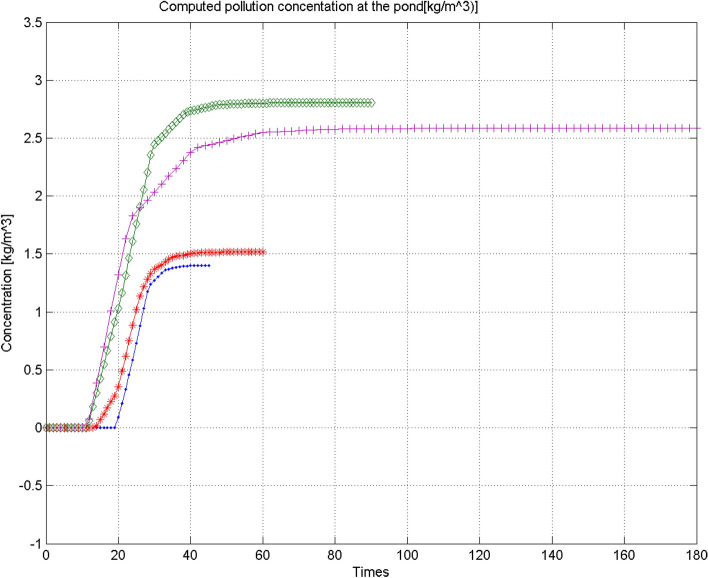
Computed pollution concentration at the pond for different final times ($t_f=45, t_f=60, t_f=90$ and $t_f=180$ days).

On Table 1 and on Fig. 6, we show the injected concentration recovered values for different period ($t_f=45, t_f=60, t_f=90$ and $t_f=180$ days) and note that when the ratio $\frac{t_f}{t_1}$ increases the parameter recovering is hard.

**Table 1:** Recovered injected concentration for different final time with $t_1=25$ days and $l_1=5$ days ($C_s{exact}=1\, {\text {kg/m}}^3$).

$\frac{t_f}{t_1}$	1.8	2.4	3.6	7.2
$t_f [days]$	45	60	90	180
Computed $C_s \,[{\text {kg/m}}^3]$	0.964	0.953	1.072	1.29
Relative error (%)	3.6	4.7	7.2	29

On Table 2 we give the different point-source location identification. We note that the recovering with stationary flow reciprocity (Eq. (14)) is better than the one with reciprocity principle applied to the transport problem (Eqs. (12), (13)). This is due to the presence of time integration in the two last equations which lead to numerical errors.

**Table 2:** Point-source location identification with stationary and transient equations.

	$x_p[m]$	$y_p[m]$
Exact value	2745	4270
Computed value with Eq. (14)	2741	4198
Relative error (%)	0.12	1.67
Computed value with Eqs. (12), (13)	2484.3	3952.9
Relative error (%)	9.49	7.42

### Pollution source identification with few observation points

In this section, we perform the identification methodology with the sole knowledge of some ’measurements’ for the hydraulic heads at the initial time (as we consider a stationary flow) and for the concentration at different times. Then, hydraulic heads and concentrations on the domain and on its boundaries are obtained by kriging^31^.

Kriging is an interpolation process that produces an optimum, linear, and unbiased estimate of the property under examination based on the available data, with the least amount of error. The advantages of kriging over more traditional interpolation methods are that kriging integrates the spatial structure of the data in the form of a variogram model in its estimate process. Moreover, it is an exact interpolator because the surface created goes across the experimental points (unless a nugget effect is incorporated). This is why Kriging interpolation has been used in hydrogeology for many years, since the work of Delhomme^32^, to estimate hydraulic parameters throughout a complete domain, optimize recognitions, simulate interfaces, and so on.

**Figure 7:**
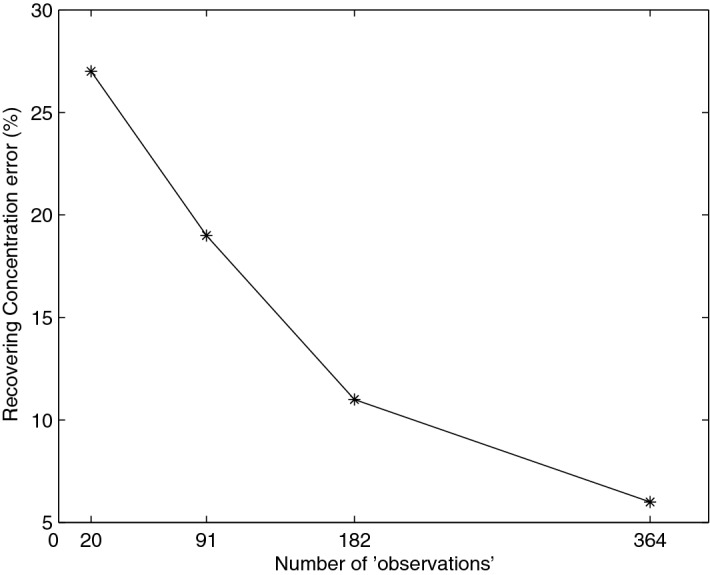
Recovering concentration’s error for different number of observations.

With interpolated heads, the resulting point-source position remains close to their reference at around 10% (identified location is (2489.7 m, 4470.7 m)). For the injected concentration identification with kriged concentrations, the error values are shown in Table 3 and on Fig. 7, for different number of retained observations. We note that as expected the error increases when the number of observations decreases until it reaches 27% for only 20 observations.

**Table 3:** Recovering concentration’s error for different number of observations ($t_1=45$ days, $t_f=25$ days and $l_1=5$ days).

Observations’ number	364	182	91	20
Computed $C_s[{\text {kg/m}}^3]$	0.964	0.89	1.19	1.27
Relative error (%)	3.6	11	19	27

### Noise sensibility pollution source identification

In actual field cases, errors may affect the collected data. There are many reasons for these errors, including the limited accuracy of equipment and the influence of sampling conditions. The quality and credibility of a given measurement depends on these errors, which must be properly identified and evaluated. So sensitivity to noise is performed on the last problem.

We have tested the effect of different levels of noise on the identification process, by adding an uniform white noise, with zero mean, to the pollution concentration measurement . The noise levels from 2 to 8% that we tested, are of the same orders of magnitude as those found in similar studies (between 5 and 10%^33^).

Table 4 shows the relative errors for different noise levels in the case with complete data. We note that the error remains acceptable (max.23%) until 8% of noise.

**Table 4:** Recovered injected concentration for different noise’s level.

Noise level (%)	0	2	4	6	8
Computed $C_s[{\text {kg/m}}^3]$	1.01	0.98	1.043	0.874	0.77
Relative error (%)	1	2	4.3	12.6	23

### Multiply point-source pollution identification

In this case, we consider that in additional to the pond, pumping wells are also point-source pollution. We change the volumetric flux’s sign for the wells and affect them the following pollution concentration: $C_s^1=0.5~{\text {kg/m}}^3$, $C_s^2=0.1~{\text {kg/m}}^3$ and $C_s^3=0.5~{\text {kg/m}}^3$ (exact positions and concentrations ares summarized on Table 5). So the studied inverse problem is to identify the four concentrations from the knowledge of the cumulative mass flux through the boundary at any time in the considered interval and the concentration on all the domain at the final time considered. We identify the positions concentrations by using the reciprocity principle respectively with the darcean equations and the advection–diffusion. As shown on Table 6, positions as well as concentrations are identified with a satisfying errors which don’t exceed $8\%$.

**Table 5:** Exact point-sources’ position and concentration in the case of 4 sources.

	$S_1$	$S_2$	$S_3$	$S_4$
*S*(*m*)	(915, 1220)	(2135, 1220)	(3355, 1220)	2747, 4270)
$C_s[{\text {kg/m}}^{3}]$	0.5	0.1	0.5	1.0

**Table 6:** Computed wells position and flux from over-specified boundary data and 20 interior observations in the case of 4 sources.

	$S_1$	$S_2$	$S_3$	$S_4$
Computed *S* (*m*)	(885, 1195)	(2096, 1197)	(3398, 1190)	2637, 4241)
Computed $C_s[kg/m^3]$	0.494	0.09	0.492	0.82
$\epsilon _S ~(\%)$	2.5	1.8	1.6	2.2
$\epsilon _f ~(\%))$	1.2	1.8	1.6	0.01

## Conclusion

In this study, we developed and applied the idea of reciprocity, which is taken from the field of mechanics and is generally applicable to linear problems, to the identification of pollution point sources in the advection–diffusion equation for the transport of solutes in aquifers.

This theory generates a strong link between the forcing terms and the resulting fields for two different forcing sets in the example of pollution transport in a Darcian flow, as illustrated in this study.

We have limited the scope of our work to the conservative transport of contaminants in the context of this research and for the sake of relevance and simplification. However, situations involving adsorption and/or degradation can be established. Furthermore, we believe that the method described here may be easily integrated into hydrogeological modeling codes such as SUTRA, MODFLOW, FEFLOW, and others, which are now widely used by water resource and environmental managers all over the world. The outcomes of the identification technique are satisfactory even with little information and available data on the state of aquifer contamination.

The simplicity of the subsequent identification process is indeed the main attractiveness of the reciprocity principle. Since the computations are limited to solving small linear equations where coefficients are given by some numerical evaluation of integrals, the computational cost of this direct method is quite minimal. The main drawback is the large amount of data required, which includes full information of concentration and fluxes at the boundaries and interfaces (which may not be available). However, as shown in Ref.^34^, using incomplete data sets is also still possible.

Other advanced parameter identification methods can be utilized in conjunction with these basic direct identification methods. They can serve as a preliminary assessment before embarking on more expensive field and laboratory investigations. These can also be effective persuasion techniques for decision-makers and competent authorities.
